# Comparisons of perventricular device closure, conventional surgical repair, and transcatheter device closure in patients with perimembranous ventricular septal defects: a network meta-analysis

**DOI:** 10.1186/s12893-020-00777-w

**Published:** 2020-05-26

**Authors:** Dongxu Li, Xu Zhou, Mengsi Li, Qi An

**Affiliations:** 1 Department of Cardiovascular Surgery, West China Hospital, Sichuan University, No. 37 Guo Xue Xiang, Chengdu, Sichuan 610041 P.R. China; 2 Evidence-based Medicine Research Center, School of Basic Medical Sciences, Jiangxi University of Traditional Chinese Medicine, Nanchang, Jiangxi P.R. China; 3 Department of Anesthesiology, West China Hospital, Sichuan University, Chengdu, Sichuan P.R. China

**Keywords:** Ventricular septal defect, Conventional surgical repair, Transcatheter device closure, Perventricular device closure, Network meta-analysis

## Abstract

**Background:**

Treatments for perimembranous ventricular septal defects (pmVSD) mainly include conventional surgical repair (CSR), transcatheter device closure (TDC), and perventricular device closure (PDC). We aimed to perform a network meta-analysis to compare the three approaches in patients with pmVSD.

**Methods:**

We searched for comparative studies on device closure and conventional repair for pmVSD to April 2020. A network meta-analysis was performed under the frequentist frame with risk ratio and 95% confidence interval. The main outcome was the procedural success rate. Additional outcomes were postoperative complications, including residual shunt, intra-cardiac conduction block, valvular insufficiency, incision infection, and pericardial effusion.

**Results:**

Twenty-four studies of 8113 patients were included in the comparisons. The pooled estimates of success rate favored the CSR compared with the PDC. No significant differences of success rate were found in the TDC versus CSR and the PDC versus TDC. The pooled estimates of incidences of the residual shunt, new tricuspid regurgitation, incision infection, and pericardial effusion favored the PDC compared with the CSR. There were no significant differences between the PDC and TDC approaches in all outcomes except new aortic regurgitation.

**Conclusion:**

The PDC technique not only reduces the risk of significant complications compared with the CSR, but also produces not inferior results compared with the TDC in selected pmVSD patients.

**PROSPERO registration number:**

CRD42019125257.

## Background

Isolated ventricular septal defects (VSDs) are the most common congenital heart diseases accounting for 20–30% of all congenital cardiac malformations [1, 2]. These defects are subdivided into different types based on their location. Up to 70–80% of VSDs are perimembranous VSDs (pmVSD); approximately 5–7% are doubly committed subarterial VSDs; approximately 5% are inlet VSDs; and the last approximately 10–15% are muscular VSDs (mVSD) which can further be classified [3].

Since over 60 years ago, Lillehei et al. firstly reported a successful repair in a patient, surgical closure on cardiopulmonary bypass had been the preferred therapeutic option for many decades [4, 5]. However, open-heart surgical repair requires cardiopulmonary bypass as well as total sternotomy, which is physically and psychologically traumatic, especially for pediatric patients [6]. Under this circumstance, devices became available that can be delivered through a percutaneous or perventricular approach to close these defects without cardiopulmonary bypass, especially in mVSD and pmVSD [7, 8].

Although many studies have confirmed the safety and efficacy of devices in the closure of isolated mVSD through a percutaneous or perventricular approach [9–13], the results of device closure for pmVSD have always been controversial with concerns including the success rate and various major and minor complications [14]. Whether the non-directly visible and limited manipulation could affect the success rate of closure, and whether the implantation of the metallic occluder device in the membranous septum in a VSD could increase the risks of residual shunt, intra-cardiac conduction block, and the valvular insufficiency [15, 16].

Most studies just compared perventricular device closure (PDC) with conventional surgical repair (CSR), or compared transcatheter device closure (TDC) with the CSR [11, 12]. The results of comparisons among the three approaches for the treatments of pmVSD are unclear.

Therefore, we aimed to perform a network meta-analysis involving direct and indirect comparisons to determine the efficacy and safety of the three approaches in pmVSD and to supply evidence in clinical treatment.

## Methods

### Search strategy

This network meta-analysis was conducted according to the Preferred Reporting Items for Systematic Reviews and Meta-Analyses extension statement (**Supplementary File** **1**) [17]. It was registered on PROSPERO international prospective registry of systematic reviews (CRD42019125257). The detailed methods could be found in a published protocol [18]. A literature search of the PubMed, EMBASE, Clinical Trials, Cochrane Library, and China National Knowledge Infrastructure was conducted for the keywords “ventricular septal defect” and “closure” until April 1, 2020, in English and Chinese. The detailed search strategies are shown in **Supplementary File** **2**. References within the retrieved articles were also analyzed. Studies were selected based on a review of the title and abstract by two independent reviewers (Li and Zhou).

### Inclusion and exclusion criteria

Studies were considered for inclusion if they presented the baseline characteristics of patients and provided original data for dichotomous and continuous variables or had sufficient information to calculate these data. Studies were selected using the following inclusion criteria: 1) two- or three-arm studies that reported at least two approaches among the CSR, TDC, and PDC; 2) studies that reported patients with pmVSD; 3) studies that described at least one variable defined as follows: a) the procedural success rate, with or without reasons for failures, b) the procedural-related complications, including a residual shunt, heart conduction block, new-onset valvular insufficiency, pericardial effusion, incision infection, and death, and c) complications at follow up [18]. In-hospital outcomes were assessed up to 30 days after the procedure. Case reports and reviews without complete information were excluded. Studies with overlapping or insufficient data were excluded. Studies that did not report patients with clear VSD anatomy were excluded. Studies that only reported patients with doubly committed subarterial VSDs or other types of VSD were excluded. When there were multiple studies from the same authors or institutions at the same period, only the largest study was included to avoid duplication of patients.

### Study quality and level of evidence

The level of evidence of the included studies was categorized by the criteria of the Center for Evidence-Based Medicine in Oxford, United Kingdom [19]. A study with a score ≥ 3b was considered to be of high quality. The methodological quality of the included studies was assessed by two authors (Li and Zhou) using the Downs and Black tool. In general, score ranges were categorized into the following four quality levels: excellent (> 25), good (20–25), fair (15–19), and poor (< 15) [20].

### Data extraction and outcomes of interest

All data were extracted independently by two authors (Li and Zhou) according to a pre-specified protocol [18]. A standardized data collection form was used to extract the data included characteristics of studies, patient baselines, the procedural success rate with reasons of failure, main complications mentioned above, and follow-up data. Successful implantation was defined as a correct device placement at a satisfactory position when confirmed by echocardiography. Residual shunts included all color jets observed across the VSD after the device placement. Conduction block included right bundle branch block (RBBB), second- or third-degree atrioventricular blocks (AVBs). Valvular insufficiency included device-related aortic or tricuspid regurgitation with the exclusion of transient early lesions that disappeared in the post-deployment period [18].

### Statistical analysis and meta-analysis

A network meta-analysis of the comparisons among the PDC, CSR, and TDC was performed with risk ratio (RR) and 95% confidential interval (CI) under the random-effects model [21]. All statistical evaluations were performed assuming a two-sided test at 5% level of significance, using Stata software (version 14.0; Stata Corp., College Station, TX, USA) with “network” command under frequentist-frame [22]. Consistency and inconsistency tests were conducted [23]. When there were significant differences among the three approaches in a parameter, the probability of best treatment was shown by “rankogram” command [22]. Funnel plots stratified by different comparisons were drawn to test the publication bias.

## Results

### Study selection

**Fig. 1:**
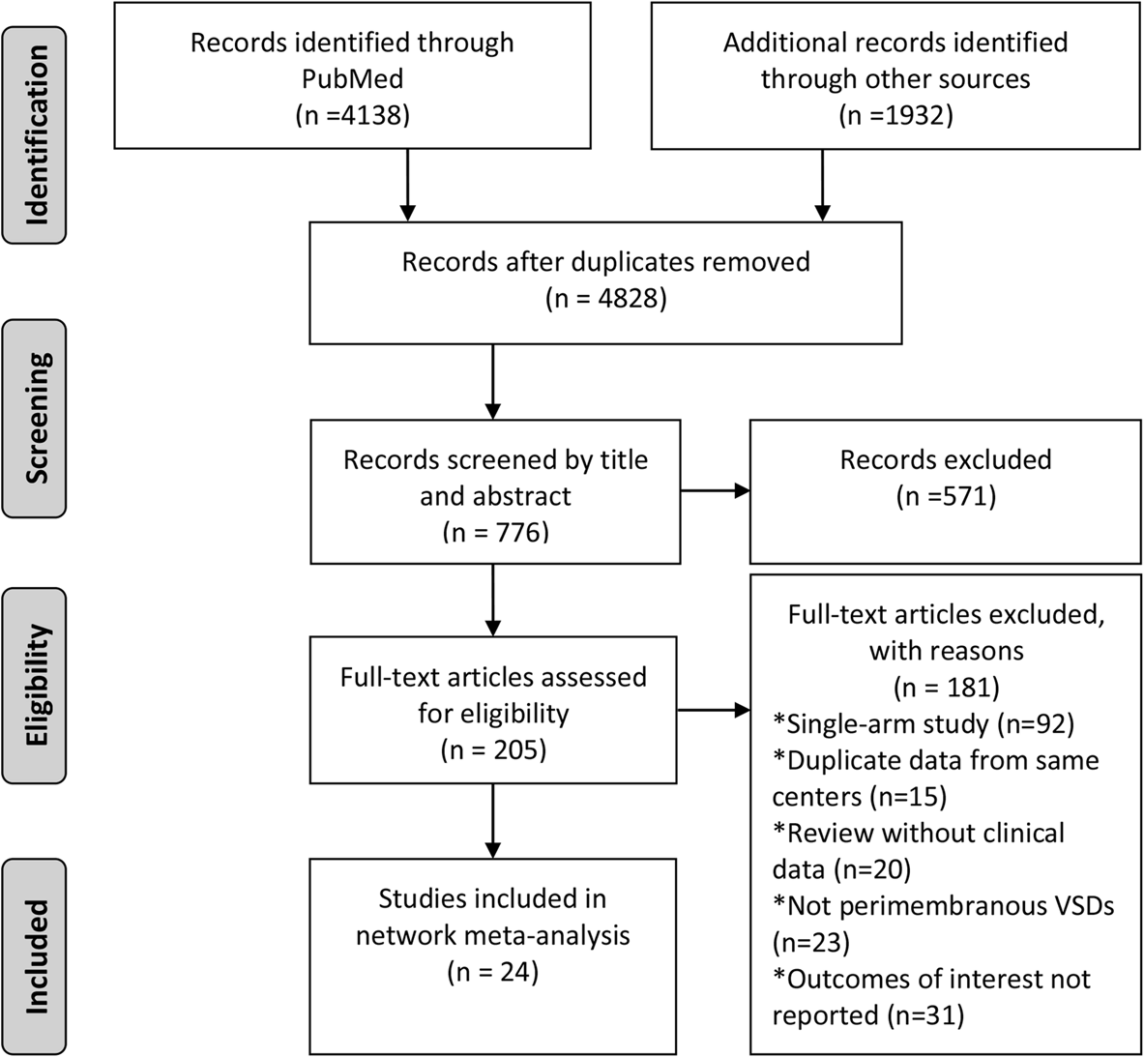
Flow diagram of included studies

A total of 24 studies involving 8113 patients were included in comparisons (PDC = 2252, CSR = 3753, TDC = 2108) [9–11, 24–44]. The flow diagram (Fig. 1) shows the detailed literature search steps. There were one two-arm study reported the direct comparison of the TDC versus PDC, ten two-arm studies reported the PDC versus CSR, 11 two-arm studies reported the TDC versus CSR, and two three-arm studies reported the comparisons of the three approaches. The characteristics of the individual studies and patient baselines are presented in Table 1 and **Supplementary Table****1**.

**Table 1:** Characteristics of included studies

Study	Region	Design	Study quality*	Anatomy (percentage of pmVSD, %)	Manufacturer of device	Device size (mm) †	Inclusion criteria	Group	Age (yr) †	Weight (kg) †	Defect size (mm) †
**PDC vs. CSR**											
Chen 2013 [24]	China	PCS	2b, 18	100	SHSMA	8.8 ± 1.8	Isolated pmVSD, 5–12 mm in size	PDC	13.3 ± 11.9	34.2 ± 16.8	7.6 ± 1.6
								CSR	13.7 ± 13.5	34.6 ± 18.5	
Hu 2014 [25]	China	PCS	2b, 20	100	SHSMA	8.4 ± 2.1	Isolated pmVSD ≤10 mm, no more than mild AR	PDC	3.7 ± 2.3	16.7 ± 7.5	7.0 ± 2.0
								CSR	1.4 ± 0.6	15.6 ± 5.0	6.8 ± 2.1
Hu 2015 [26]	China	RCS	2b, 17	100	Lifetech Med	7.6 ± 2.0	Isolated pmVSD ≤10 mm, no AR	PDC	5.3 ± 3.6	18.1 ± 10.4	5.1 ± 2.2
								CSR	4.4 ± 3.0	16.4 ± 6.1	4.2 ± 1.8
Luo 2015 [27]	China	RCS	2b, 17	100	SHSMA	NA	Isolated pmVSD, no severe PAH	PDC	3.7 ± 5.5	14.9 ± 13.0	4.5 ± 1.6
								CSR	3.7 ± 2.4	15.9 ± 5.3	4.6 ± 2.4
Voitov 2017 [28]	Russia	RCT	1b, 22	81.9	SHSMA	4–11	Isolated VSD ≤10 mm, no more than mild AR	PDC	2.9 ± 0.4	13.9 ± 1.2	5.3 ± 0.3
								CSR	3 ± 0.4	14.5 ± 1.2	6.2 ± 0.5
Xing 2015 [29]	China	RCS	2b, 19	86.9	AGA, SHSMA and Lifetech Med	6.5 ± 5.3	Restrictive isolated VSD, no severe PAH	PDC	1 ± 0.6	9.8 ± 5.9	5.2 ± 3.0
								CSR	0.9 ± 0.7	8.6 ± 7.8	6.8 ± 3.8
Xu 2012 [30]	China	PCS	2b, 19	100	SHSMA	4–18	Isolated pmVSD, 3–12 mm, no more than mild AR, age < 2 years	PDC	0.7 ± 0.3	9.8 ± 1.8	5.1 ± 1.5
								CSR	0.5 ± 0.2	7.7 ± 1.9	6.4 ± 1.7
Yao 2013 [31]	China	RCS	2b, 12	70.9	SHSMA	NA	Isolated VSD, no more than severe PAH	PDC	4.7 ± 0.4	18.4 ± 1.0	NA
								CSR	4.6 ± 0.5	17.7 ± 1.2	NA
Liu 2018 [32]	China	RCT	1b, 26	100	Lifetech Med	8.9 ± 1.4	isolated pmVSD with 3 to 10 mm, normal or mild PAH	PDC	2 ± 0.7	13.2 ± 1.9	5.5 ± 1.4
								CSR	2 ± 0.7	13.2 ± 1.9	5.5 ± 1.4
Chen 2018 [33]	China	RCS	2b, 17	100	Lifetech Med and Shandong Visee	6–14	pmVSD< 10, with no AR	PDC	1.3 ± 1.2	8.3 ± 2.6	4.2 ± 1.1
								CSR	1.2 ± 1.1	8.1 ± 2.5	5.1 ± 1.2
**TDC vs. CSR**											
Yang 2014 [9]	China	RCT	1b, 24	100	SHSMA	7.4	Age 3–12 years, Weight > 10 kg, VSD > 3 mm	TDC	5.5 ± 2.6	22.1 ± 13.8	5.2 ± 6.1
								CSR	5.8 ± 2.4	20.5 ± 12.4	5.9 ± 5.3
Liu 2012 [10]	China	RCS	2b, 18	100	SHSMA	6.2 ± 2.3	Age > 3 years, Weight > 10 kg, VSD < 16 mm	TDC	18.1 ± 15.1	NA	4.1 ± 1.4
								CSR	7.5 ± 9.4	NA	6.3 ± 4.1
Pawelec-Wojtalik 2005 [11]	Poland	RCS	2b, 15	100	AGA	6.4 ± 2.2	Weight > 5 kg	TDC	7.8 ± 6.4	28.1 ± 21.3	6.2 ± 1.1
								CSR	2.6 ± 2.3	16.7 ± 8.4	4.8 ± 0.9
Cheng 2007 [34]	China	PCS	2b, 18	100	AGA	NA	Weight > 10 kg, VSD < 12 mm	TDC	7.5 ± 2.4	NA	4.8 ± 2.3
								CSR	4.4 ± 2.5	NA	8.2 ± 2.3
Zheng 2009 [35]	China	RCS	2b, 17	77.8	AGA	NA	Age > 2.5 years, Weight > 11 kg, VSD 3–12 mm	TDC	2.5–15.5	11–63.5	3–12
								CSR	2.8–52.5	11.5–68	3–36
Oses 2010 [36]	Canada	RCS	2b, 16	100	AGA	6.4 ± 2.2	VSD size < 6 mm, no age or Weight limit. VSD size > 6 mm, age > 6 years or Weight > 6 kg	TDC	9.1 ± 5.1	NA	9.4 ± 3.9
								CSR	1.8 ± 3.6	NA	
Chen 2014 [37]	China	RCS	2b, 16	100	SHSMA	6.5 ± 2.1	Isolated pmVSD	TDC	16 ± 11.7	41.3 ± 18.5	4.1 ± 1.2
								CSR	3.8 ± 2.4	15.3 ± 5.3	4.3 ± 1.3
Zhu 2007 [38]	China	RCS	2b, 16	100	AGA, SHSMA	8.1 ± 2.5	NA	TDC	11 ± 8	33 ± 17	4.4 ± 1.1
								CSR	3–51	33 ± 16	5.2 ± 1
Chen 2015 [39]	China	RCS	2b, 17	100	NA	NA	Age > 2 years, Weight > 8 kg, VSD < 14 mm	TDC	5 ± 1.2	16.5 ± 4.5	4.3 ± 1.7
								CSR	4.9 ± 1.4	16.2 ± 5	5.4 ± 1.6
Xiao 2008 [40]	China	RCS	2b, 15	100	AGA	NA	Age > 3 years, Weight > 5 kg, VSD < 12 mm	TDC	11.2 ± 6.8	NA	5.6 ± 1.3
								CSR	12 ± 7.6	NA	6.1 ± 1.8
Chen 2010 [41]	China	RCS	2b, 16	94.9	Lifetech Med	NA	Age > 3 years, Weight > 10 kg, VSD < 10 mm	TDC	10.6 ± 11.1	NA	5.5 ± 2.4
								CSR	8.3 ± 7.9	NA	7.4 ± 6
**TDC vs. PDC**											
Huang 2019 [42]	China	RCS	2b, 16	100	Lifetech Med and AGA	6.6 ± 2.8	isolated VSD with no AR	TDC	6.2 ± 6.3	33.1 ± 21.5	5.9 ± 2.3
								PDC	6.5 ± 5.2	31.5 ± 23.4	6.1 ± 2.1
**PDC vs. TDC vs. CSR**											
Fang 2018 [43]	China	RCS	2b, 17	100	Shandong Visee	6–14	isolated VSD with no PAH	PDC	1.6 ± 1.3	10.1 ± 2.3	5.3 ± 1.1
								TDC	2.1 ± 0.8	10.6 ± 2.8	5.1 ± 1
								CSR	1.4 ± 1.5	9.5 ± 3.1	5.9 ± 1.1
Chen 2019 [44]	China	RCS	2b, 17	100	Shandong Visee and AGA	4.8 ± 1.4	restrictive pmVSD with no severe PAH	PDC	2.7 ± 1.5	18.8 ± 2.3	4.3 ± 0.7
								TDC	2.9 ± 0.7	19.7 ± 3.9	4.2 ± 0.9
								CSR	2.7 ± 1.1	19.3 ± 3.0	4.4 ± 0.8

*PDC* perventricular device closure; *TDC* transcatheter device closure; *CSR* conventional surgical repair; *PCS* Prospective cohort study; *RCS* retrospective cohort study; *VSD* ventricular septal defects; *pmVSD* perimembranous VSD; *yr* year; *mm* millimeter; *RCT* Randomized controlled trial; *NA* not available; *SHSMA* Shanghai Shape Memory Alloy Co., Ltd.; *AGA* AGA Medical Corporation; *Lifetech Med* Lifetech Scientific Co., Ltd.; *Shandong Visee* Shandong Visee Medical Devices Co., Ltd.; *AR* aortic regurgitation; *PAH* pulmonary artery hypertension; *PDC* perventricular device closure; *TDC* transcatheter device closure; *CSR* conventional surgical repair; *Studies were categorized by Evidence-based Medicine Levels of Evidence and assessed by Downs and Black scoring system [19, 20]; †Continuous variables were presented as mean ± standard deviation or median (interquartile range) or just range

The study periods were from 2007 to 2018. Three randomized controlled trials (RCTs) were included, with two studies involving the PDC versus CSR and one involving the TDC versus CSR. Besides, there were four prospective and 17 retrospective cohorts. Most of the studies were published from China, just one from Russia, one from Poland, and one from Canada. Five studies were published in Chinese, and the others were in English. The level of evidence and quality score of included studies are shown in Table 1.

Nineteen of the 24 included studies were patients all with pmVSD. In the remaining five studies, patients with pmVSD accounted for more than 70%. According to these studies, the mean age of patients was 0.5–15, 0.7–15, and 2.1–18.1 years old in the CSR, PDC, and TDC groups, respectively; and the mean weight of patients in 3 groups were 7.7–34.6, 8.3–34.2, and 10.6–41.3 kg. Additionally, the defect size of patients underwent PDC was reported less than 10 mm in most included studies (Table 1).

### Outcomes of comparisons

**Fig. 2:**
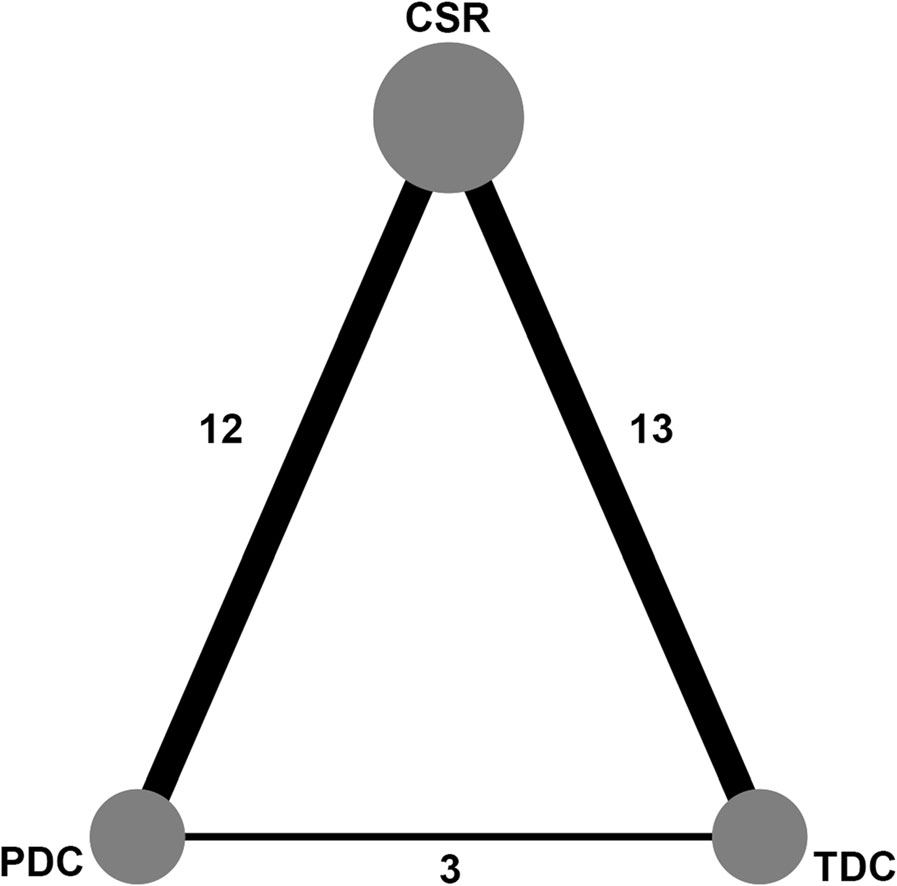
Network plot of included studies

**Fig. 3:**
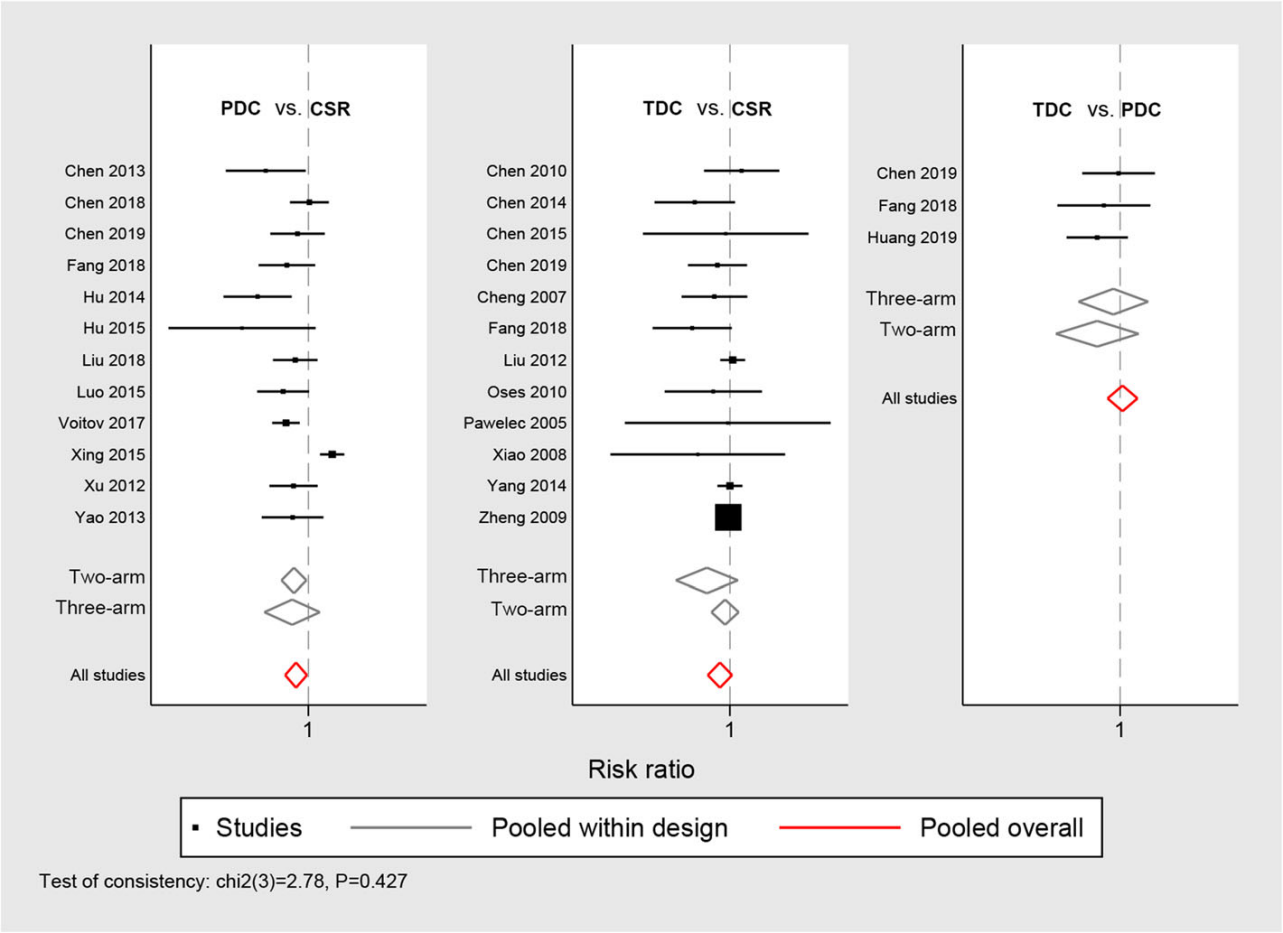
Forest plot of the success rate; the pooled estimates showed significant differences between PDC versus TDC, but no significant differences in TDC versus CSR and TDC versus PDC. PDC perventricular device closure; TDC, transcatheter device closure; CSR, conventional surgical repair

The main results of the comparisons are summarized in Fig. 2**,** Fig. 3 and Table 2. Moreover, other forest plots are shown in **Supplementary Fig.** **1****–****12**. The *P*-value for the inconsistency of some parameter was not available where there was no direct comparison between the PDC and TDC techniques.

**Table 2:** Results of comparisons with tests of inconsistency

Title/Subtitle	PDC versus CSR			TDC versus CSR			TDC versus PDC			P for inconsistency
	RR	95% CI	P	RR	95% CI	P	RR	95% CI	P	
Perioperative indexes										
Procedural success rate	0.98	0.96–0.99	0.03	0.98	0.97–1.00	0.11	1.00	0.98–1.02	0.75	0.42
Residual shunt	0.61	0.40–0.93	0.02	0.79	0.51–1.22	0.29	1.29	0.73–2.29	0.38	0.19
II degree AVB	1.52	0.61–3.81	0.36	0.59	0.25–1.42	0.24	0.39	0.11–1.32	0.13	0.91
Complete AVB	0.90	0.43–1.88	0.78	0.98	0.46–2.10	0.96	1.08	0.42–2.82	0.86	0.40
RBBB	0.53	0.30–0.94	0.03	0.36	0.21–0.62	< 0.01	0.68	0.31–1.47	0.33	NA
Procedure–induced AR	0.53	0.28–1.01	0.05	1.30	0.63–2.69	0.47	2.45	1.33–4.49	< 0.01	0.32
Procedure–induced TR	0.55	0.33–0.92	0.02	0.56	0.19–1.66	0.29	1.04	0.34–3.19	0.95	NA
Incision complications	0.50	0.26–0.96	0.04	0.39	0.13–1.20	0.10	0.77	0.23–2.61	0.67	0.95
Pericardial effusion	0.47	0.29–0.76	< 0.01	NA	NA	NA	NA	NA	NA	NA
Follow–up data										
Residual shunt	0.25	0.14–0.42	< 0.01	0.29	0.11–0.79	0.02	1.19	0.38–3.68	0.78	NA
RBBB	0.50	0.34–0.72	< 0.01	0.38	0.27–0.55	< 0.01	0.77	0.46–1.30	0.33	NA
Procedure–induced AR	0.72	0.19–2.72	0.62	1.52	0.27–8.51	0.64	2.12	0.36–12.42	0.40	0.49
Procedure–induced TR	0.31	0.19–0.48	< 0.01	0.48	0.09–2.50	0.39	1.58	0.29–8.69	0.60	NA

*PDC* perventricular device closure; *CSR* conventional surgical repair; *TDC* transcatheter device closure; *RR* relative risk; *NA* not available; *AVB* atrioventricular block; *RBBB* right bundle branch block; *AR* aortic regurgitation; *TR* tricuspid regurgitation

In terms of procedural success rate (Fig. 3), the pooled estimates of the success rate favored the CSR group (RR: 0.98, 95%CI: 0.96–0.99, *p* = 0.03) when compared with the PDC group, whereas no difference was found in the TDC versus CSR (RR: 0.98, 95%CI: 0.97–1.00, *p* = 0.11) and the TDC versus PDC (RR: 1.00, 95%CI: 0.98–1.02, *p* = 0.75).

With respect to complications, the pooled estimates of the incidence of residual shunt (RR: 0.61, 95%CI: 0.40–0.93, *p* = 0.02), RBBB (RR: 0.53, 95%CI: 0.30–0.94, *p* = 0.03), new TR (RR: 0.55, 95%CI: 0.33–0.92, *p* = 0.02), incision complications (RR: 0.50, 95%CI: 0.26–0.96, *p* = 0.04), and pericardial effusion (RR: 0.47, 95%CI: 0.29–0.76, *p* < 0.01) favored the PDC group than the CSR group. No significant differences were found in second degree AVB (RR: 1.52, 95%CI: 0.61–3.81, *p* = 0.36), complete AVB (RR: 0.90, 95%CI: 0.43–1.88, *p* = 0.78), and new AR (RR: 0.53, 95%CI: 0.28–1.01, *p* = 0.05) were found between the PDC and CSR groups. Additionally, the risk of RBBB (RR: 0.36, 95%CI: 0.21–0.62, *p* < 0.01) was lower in the TDC group than in the CSR group. Other synthetic results in Table 2 show there were no differences in complications between the PDC and TDC groups except new AR (RR: 2.45, 95%CI: 1.33–4.49, *p* < 0.01).

During the follow-up (Table 2), the pooled estimates of the incidence of residual shunt (RR: 0.25, 95%CI: 0.14–0.42, *p* < 0.01; RR: 0.29, 95%CI: 0.11–0.79, *p* = 0.02) and RBBB (RR: 0.50, 95%CI: 0.34–0.72, *p* < 0.01; RR: 0.38, 95%CI: 0.27–0.55, *p* < 0.01) favored device groups than conventional group. However, no differences were observed in parameters above between the PDC and TDC groups. Additionally, there were no significant differences in new AR and TR among the three approaches.

### Publication bias

**Fig. 4:**
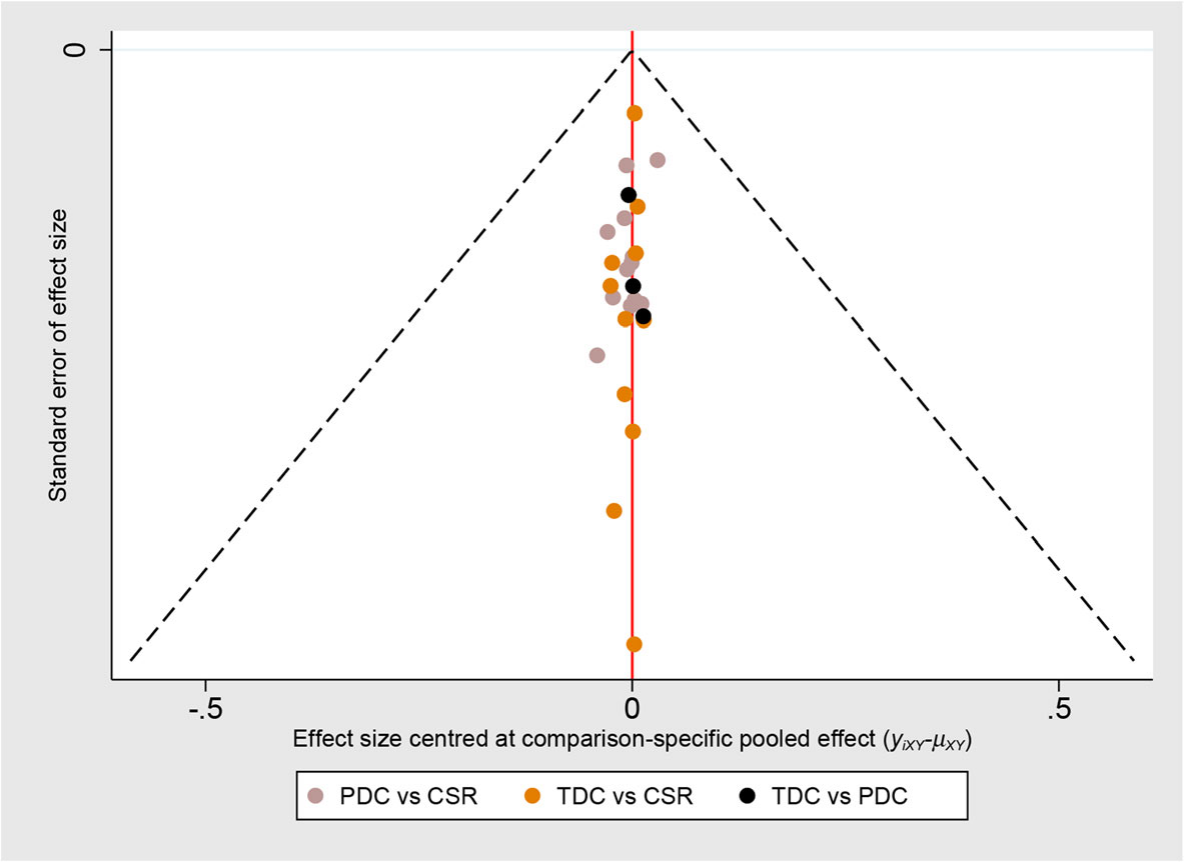
Funnel plot of success rate; the funnel plot showed symmetrical distributions, suggesting that there was no evidence of publication bias in this pooled estimate. PDC, perventricular device closure; TDC, transcatheter device closure; CSR, conventional surgical repair

Funnel plot of success rate was performed (Fig. 4). Moreover, no significant asymmetry was found, which suggested that there was no evidence of publication bias in this network meta-analysis among the studies included.

## Discussion

As a standard, the CSR could be performed in almost congenital VSD patients with cardiopulmonary bypass. In contrast, the TDC approach could be minimally invasive but is limited by the position of defect, vascular access and the radiation [10]. In this circumstance, the PDC technique was introduced and confirmed safe and efficacious in more selected patients with minimal invasion and non-radiation [2].

According to our inclusion criteria, patients in most included studies were all with pmVSD. Although in some studies, a small number of the patients were with other types of VSD, more than 70% were pmVSD patients [25, 26, 29, 35, 41]. In the included comparative studies, patients with pmVSD underwent TDC were generally above 2 years old or above 10 kg. At the same time, patients underwent PDC were generally above 0.7 years old or more than 8 kg. In our center, most patients underwent PDC were more than 1 year old or at least 10 kg [2]. In most cases, the defect size in patients underwent device closure should not be more than 10 mm [45]. Additionally, under these conditions, such as related aortic regurgitation or more than moderate pulmonary hypertension, device closure should not be performed in patients with pmVSD [22–26].

Furthermore, the PDC even was reported to be performed in patients less than 1 year old or patients with doubly committed subarterial VSDs [46, 47]. However, due to the limited number of reported studies, the safety and efficacy of this technique in such patients are unclear.

Since firstly reported in non-muscular VSD [4, 48], the PDC approach has widely spread across China [49]. Furthermore, Schreiber et al. in Germany and Omelchenko et al. in Russia reported cases that underwent such a technique [50, 51]. Even so, Wells suggested that there should be multicenter data with long-term follow-up to confirm the risk and efficacy of this technique [52]. After that, Voitov et al. in Russia and Liu et al. in China performed an RCT to compare the PDC and CSR, respectively [28, 43].

Although concerns exist about the use of device closure in congenital VSDs, studies have suggested no significant differences in procedural success rate and postoperative complications between the TDC and CSR [12]. With the same delivery apparatus and device equipment, the PDC technique has the advantage of direct access and easy manipulation, which maybe makes it possible to avoid the valves and conductions in more selected patients [5, 14].

Although the success rate was reported at 96.6 and 98.0% for the PDC by Voitov et al. and Liu et al., it was still lower than the rate of approximately 100% for the CSR [28, 43]. As to the TDC versus CSR, Yang et al. and Saurav et al. suggested that there was no difference in success rate between the two groups [9, 12]. And a three-arm study by Fang et al. also demonstrated that PDC had a comparable success rate with the TDC technique [43].

To some degree, operation and recovery time are associated with the surgical wound. But Luo et al. showed no significant differences in ventilation time, ICU stay, and hospital stay between the PDC and CSR [27]. At the same time, three RCTs all demonstrated that operation time, ventilation time, and hospital stay were better in the device group, which were consistent with our synthetic results [9, 28, 32]. Moreover, it is not necessary to intubate in patient with the TDC treatment in most situations. But Oses et al. [36] still reported patients with shorter ventilation time in the TDC group than CSR group.

In terms of complications, Liu et al. reported no differences in residual shunt, complete AVB, RBBB, valvular regurgitation, incision infection, and pericardial effusion between the PDC and CSR [32]. In contrast, Voitov et al. reported no differences in AVB between two the approaches, but a lower incidence of the residual shunt in the PDC group compared with CSR [28]. Zhou et al. performed a meta-analysis of the PDC versus CSR [53]. They showed no differences in residual shunt and valvular insufficiency between the two approaches, but with a lower risk of arrhythmias in the PDC approach [53].

Yang et al. from Singapore conducted a meta-analysis of the proportion of complications in the TDC technique [54]. They demonstrated the pooled incidences of the residual shunt, complete AVB, aortic regurgitation, and tricuspid regurgitation were 25.5, 2.4, 2.0, and 1.7% respectively [54]. Compared with the CSR, Yang et al. from China and Saurav et al. both reported no significant differences in the above complications in the TDC technique [9, 12]. Similarly, Fang et al. showed no significant differences in those mentioned complications not only between the TDC and CSR, but among the three approaches [43].

Technically, direct access greatly facilitates the manipulation of device position and orientation during deployment which contributes to the lower risk of residual shunt in the PDC compared with TDC [5]. Moreover, the more or less perpendicular-angle perventricular performance with wire and sheath results in less damage to the atrioventricular conductions and valves [2].

Concerning the follow-up data, Voitov et al. reported a lower risk of the residual shunt in the PDC compared with CSR, but no significant differences in aortic and tricuspid defects between the two techniques [28]. Fang et al. revealed no significant differences in the complications among the three techniques [43]. The follow-up duration ranged from the least 0.3 to the most 3.9 years. The implanted device seems not worse than the patch.

### Study limitations

Our study had some limitations. First, most studies were from China, and this might have resulted in regional bias. Second, some included studies involving different design and patients with different VSD types might lead to heterogeneity. It was difficult to segregate different VSD types in some studies. To incorporate heterogeneity in treatment effects, we employed random-effects model and excluded studies reported patients with unclear or other types of VSD. Third, the follow-up intervals in different studies were different and no more than 5 years. Studies with long-term follow-up are needed. Fourth, because of the limited number of three-arm studies, many pooled estimates of the PDC versus TDC were just from indirect comparison without the test of inconsistency.

## Conclusion

The PDC technique not only reduces the risk of significant complications compared with the CSR, but also produces not inferior results compared with the TDC in selected pmVSD patients. The PDC technique appears to be a safe and effective option for selected patients with pmVSD.

## Supplementary information

**Additional file 1:Supplementary File 1.** PRISMA network meta-analysis checklist. **Supplementary File 2.** Search strategy. **Supplementary Table 1.** Patient baselines in subgroups of included studies. **Supplementary Fig. 1.** Forest plot of residual shunt. **Supplementary Fig. 2.** Forest plot of second degree AVB. **Supplementary Fig. 3.** Forest plot of complete AVB. **Supplementary Fig. 4.** Forest plot of RBBB. **Supplementary Fig. 5.** Forest plot of procedure–induced AR. **Supplementary Fig. 6.** Forest plot of procedure–induced TR. **Supplementary Fig. 7.** Forest plot of incision complications. **Supplementary Fig. 8.** Forest plot of pericardial effusion. **Supplementary Fig. 9.** Forest plot of residual shunt in follow-up data. **Supplementary Fig. 10.** Forest plot of RBBB in follow-up data. **Supplementary Fig. 11.** Forest plot of procedure–induced AR in follow-up data. **Supplementary Fig. 12.** Forest plot of procedure–induced TR in follow-up data.

## Data Availability

The datasets used are available from the corresponding author on reasonable request.

## Abbreviations

VSD: Ventricular septal defect
CSR: Conventional surgical repair
TDC: Transcatheter device closure
PDC: Perventricular device closure
RBBB: Right bundle branch block
AVB: Atrioventricular block
RR: Risk ratio
CI: Confidential interval
RCT: Randomized controlled trial
